# HT SpaceM enables high-throughput mapping of metabolic diversity at the single-cell level

**DOI:** 10.1016/j.crmeth.2025.101268

**Published:** 2026-01-16

**Authors:** Rune Daucke, Erwin M. Schoof

**Affiliations:** 1 Department of Biotechnology and Biomedicine, Technical University of Denmark, 2800 Kgs. Lyngby, Denmark

## Abstract

Expanding metabolomic profiling to the single-cell level can reveal metabolic heterogeneity and clinically relevant subpopulations, yet existing methods lack sensitivity and scale. To address this gap, in a recent issue of *Cell*, Delafiori and colleagues introduce HT SpaceM, a high-throughput MALDI workflow enabling sensitive, reproducible, and scalable single-cell metabolomics.

## Main text

**Figure 1:**
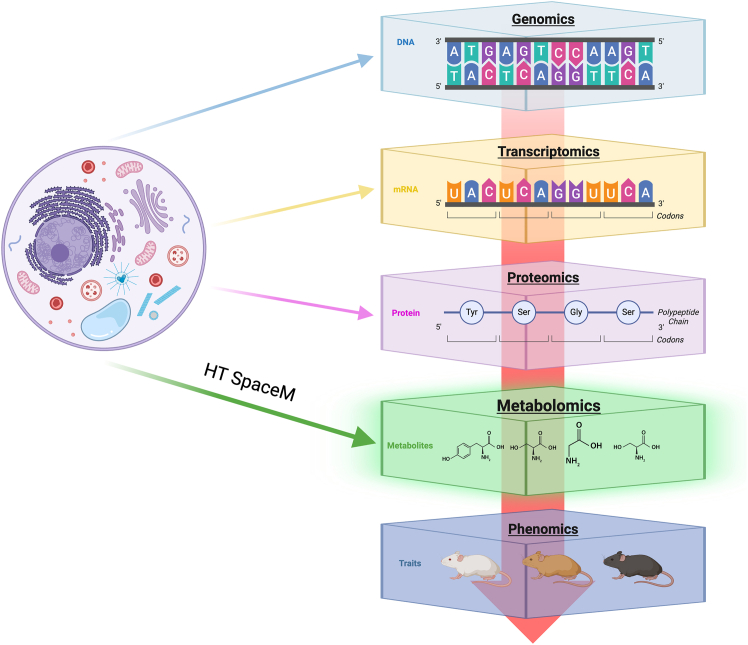
Overview of molecular information flow across omics layers DNA in the genome is transcribed into mRNA (transcriptome) and translated into proteins (proteome), which help shape the small-molecule composition of the metabolome. Together, these layers give rise to the phenotypic traits observed in organisms. At the metabolomics stage, HT-SpaceM further advances this framework by providing high-resolution spatial and molecular profiling, deepening insight into metabolic organization within tissues. Figure created with BioRender (https://biorender.com/).

Decoding cellular heterogeneity lies at the forefront of modern omics research, as it provides deeper insight into the molecular drivers of individual cells rather than population averages. Mapping the metabolome of each cell is critically important, as metabolites represent the downstream functional output of gene expression and protein activity. Yet, while single-cell (SC) transcriptomics has dominated the field for nearly two decades^1^^,^^2^ and SC proteomics has flourished in recent years,^3^^,^^4^ SC metabolomics (SCM) has yet to achieve the same level of methodological maturity.^5^ Challenges such as the extremely low abundance of many metabolites, the inability to amplify molecular content, and their structural diversity have constrained SCM to targeted analytes or a limited number of detectable features. Among the omic layers, proteomics and metabolomics sit closest to the phenotypic end of the molecular hierarchy, capturing a cell’s functional state rather than its potential.^6^ This proximity to phenotype makes metabolomics a crucial bridge between molecular expression and function, linking cellular metabolism directly to the genetic and environmental factors that govern behavior (Figure 1).

Expanding metabolomic profiling to the SC level has a profound impact across biological and clinical disciplines, particularly in cancer research, where tumor evolution, metabolic plasticity, and therapy resistance are driven by factors that other omic layers often fail to capture. Such analyses can uncover rare subpopulations with distinct metabolic phenotypes, reveal cells that escape therapy or adapt to microenvironmental stress, and map the metabolic networks that underlie these adaptive states. To accommodate needs such as these and advance the field of SCM, Delafiori and colleagues introduce High-Throughput (HT) SpaceM, a workflow designed to enable scalable, reproducible, and small-molecule-focused SCM, moving beyond existing methods that often detect only lipids or are limited to hundreds or a few thousand cells.^7^

HT SpaceM builds on the original SpaceM platform^8^ with major improvements in throughput, precision, and metabolite coverage. Custom laser-etched slides containing 40 micro-wells with fiducial markers allow up to 40 samples to be analyzed in parallel, increasing throughput 5-fold while reducing variability between slides. After cell seeding and preparation, microscopy captures the spatial arrangement of individual cells, followed by MALDI-imaging mass spectrometry to record ion distributions at SC resolution. Using an AP-SMALDI5 MALDI-imaging setup, the authors optimized ionization and acquisition parameters for small-molecule detection rather than lipid profiles. The etched grid ensures accurate alignment between microscopic cell images and MALDI sampling coordinates, enabling reliable assignment of metabolite signals to individual cells. The computational pipeline performs automatic image registration, cell segmentation, and normalization across wells and slides. Metabolite annotation is carried out through the METASPACE^9^^,^^10^ framework and validated by liquid chromatography-tandem mass spectrometry (LC-MS/MS). The framework includes quality control and statistical evaluation to assess reproducibility and detect biological variability. A network-based analysis of metabolite co-abundance identifies coordinated biochemical pathways and metabolic hubs across thousands of single cells.

Using HT SpaceM, the authors detected 135 unique ions in 78,500 cells across 72 samples; of these ions, 73 were validated as metabolites by bulk LC-MS/MS. In mixed and monoculture experiments with HeLa, NIH3T3, and NCI-H460 cells, the method resolved distinct metabolic fingerprints reflecting differences in energy metabolism and metabolite composition. Extending the analysis to nine cancer cell lines from the NCI-60 collection plus HeLa, the authors profiled over 42,000 cells and detected 202 ion features. Multivariate analysis revealed cell-line-specific metabolic signatures, with variation in amino acids, carnitine-related species, and nucleoside metabolites across cell lines. Co-abundance network analysis identified central metabolites that acted as metabolic hubs, linking multiple pathways and reflecting functional differences between cell types. SC mapping also revealed intra cell-line heterogeneity, with subpopulations displaying divergent metabolic states. These results demonstrate that HT SpaceM can scale to tens of thousands of cells while resolving metabolic diversity, identifying cell-type-specific pathways, and linking metabolite patterns to cellular phenotypes.

To test the method under metabolic perturbation, the authors inhibited glycolysis in HeLa cells with 2-deoxyglucose (2-DG) and profiled about 15,700 single cells. HT SpaceM revealed a marked metabolic shift consistent with glycolytic blockade, including glucose accumulation and decreased glycolytic intermediates, alongside elevated citric acid levels indicative of compensatory activation of the TCA cycle. Network analysis showed that glucose became a central metabolite after treatment, with expanded connectivity to other metabolites, reflecting a transient glucose-centered metabolic coordination. Over time, correlations within amino acid and energy-related metabolites changed dynamically, suggesting adaptive reorganization of cellular metabolism. Unsupervised clustering uncovered substantial heterogeneity among 2-DG-treated cells, exceeding the differences between 12- and 24-h exposures. Distinct metabolic subpopulations were driven by markers such as glutamine, AMP, fatty acids, and nucleotides, consistent with cells occupying different cell-cycle states. This heterogeneity was reproducible across replicates and independent of technical variation, demonstrating that SC metabolic diversity persists even under uniform pharmacological inhibition.

HT SpaceM represents a major step toward scalable and sensitive SCM. By combining a redesigned high-density slide format with optimized MALDI imaging and automated data processing, it enables analysis of tens of thousands of cells across multiple samples within a single experiment. Despite these advances, several challenges remain before the true power of HT SpaceM can be fully unlocked. Metabolite identification in MALDI imaging is still limited to MS1-level accuracy, leaving structural isomers unresolved and requiring bulk LC-MS/MS for confirmation. Data sparsity and ion suppression remain key technical hurdles, demanding improved acquisition sensitivity and more advanced computational strategies. The workflow is optimized for adherent cells, and adaptation to tissue sections or suspension cells will require higher spatial resolution and tailored protocols. In addition, large-scale application will depend on further automation and accessible analysis pipelines that lower the computational barrier for SCM. Nevertheless, the authors demonstrate an important technological leap by delivering a scalable, quantitatively rigorous platform for small-molecule SCM that the broader community can now adopt and extend.

In parallel with the recent surge of multi-omic studies, first combining multiple sequencing-based layers and, more recently, proteins and mRNA integration^11^ of SCM data with other omics layers is poised to have immense impact. Combining SCM with transcriptomics or proteomics could directly link gene expression programs to metabolic phenotypes, clarifying how transcriptional regulation translates into biochemical function. Extending HT SpaceM toward spatially resolved SC mapping in tissues could uncover metabolic niches within the tumor microenvironment or immune landscapes, providing essential context that dissociated-cell analyses cannot capture. As annotation coverage increases, spatial precision improves, and computational tools mature, HT SpaceM will provide both a robust experimental platform and a conceptual framework for connecting metabolism with cell state, microenvironment, and function. Together, these developments position SCM as a central component of next-generation multiomic and spatial biology.

## Declaration of interests

The authors declare no competing interests.
